# Sustained oscillations, irregular firing, and chaotic dynamics in hierarchical modular networks with mixtures of electrophysiological cell types

**DOI:** 10.3389/fncom.2014.00103

**Published:** 2014-09-02

**Authors:** Petar Tomov, Rodrigo F. O. Pena, Michael A. Zaks, Antonio C. Roque

**Affiliations:** ^1^Institute of Mathematics, Humboldt University of BerlinBerlin, Germany; ^2^Laboratory of Neural Systems, Department of Physics, School of Philosophy, Sciences and Letters of Ribeirão Preto, University of São PauloRibeirão Preto, Brazil

**Keywords:** self-sustained activity, cortical oscillations, irregular firing activity, hierarchical modular networks, cortical network models, intrinsic neuronal diversity, cerebral cortex, chaotic neural dynamics

## Abstract

The cerebral cortex exhibits neural activity even in the absence of external stimuli. This self-sustained activity is characterized by irregular firing of individual neurons and population oscillations with a broad frequency range. Questions that arise in this context, are: What are the mechanisms responsible for the existence of neuronal spiking activity in the cortex without external input? Do these mechanisms depend on the structural organization of the cortical connections? Do they depend on intrinsic characteristics of the cortical neurons? To approach the answers to these questions, we have used computer simulations of cortical network models. Our networks have hierarchical modular architecture and are composed of combinations of neuron models that reproduce the firing behavior of the five main cortical electrophysiological cell classes: regular spiking (RS), chattering (CH), intrinsically bursting (IB), low threshold spiking (LTS), and fast spiking (FS). The population of excitatory neurons is built of RS cells (always present) and either CH or IB cells. Inhibitory neurons belong to the same class, either LTS or FS. Long-lived self-sustained activity states in our network simulations display irregular single neuron firing and oscillatory activity similar to experimentally measured ones. The duration of self-sustained activity strongly depends on the initial conditions, suggesting a transient chaotic regime. Extensive analysis of the self-sustained activity states showed that their lifetime expectancy increases with the number of network modules and is favored when the network is composed of excitatory neurons of the RS and CH classes combined with inhibitory neurons of the LTS class. These results indicate that the existence and properties of the self-sustained cortical activity states depend on both the topology of the network and the neuronal mixture that comprises the network.

## 1. Introduction

The resting state of the brain, i.e., its state in the absence of sensory stimuli or behavioral tasks, shows sustained ongoing activity characterized by irregular neuronal firing and macroscopic ensemble oscillations covering a broad frequency range, from less than 1 Hz up to more than 100 Hz (Arieli et al., 1995; Bringuier et al., 1999; Tsodyks et al., 1999; Buzsáki and Draguhn, 2004; Fox and Raichle, 2007; Roopun et al., 2008; Shmuel and Leopold, 2008; Hahn et al., 2010). Experimental and theoretical work suggests that this ongoing resting state activity may have an important role to endow the brain with flexibility in dealing with diverse cognitive and behavioral situations (Lakatos et al., 2008; Gong and van Leeuwen, 2009; Lewis et al., 2009; Luczak et al., 2009; Sadaghiani et al., 2010; Destexhe, 2011; Steinke and Galán, 2011).

Since the cortex during a resting state is essentially disconnected from external stimuli, it is in a dynamic regime in which neural activity is self-sustained (Stratton and Wiles, 2010). It is important to understand the mechanisms responsible for self-sustained activity (SSA) in the cortical network: the roles of the structural organization of cortical connections as well as of the intrinsic characteristics of neurons that constitute the network.

The architecture of the cortical connections presents different features when viewed from different spatial scales. At a microscopic scale cortical circuitry is highly recurrent with both excitatory and inhibitory neurons involved in many superposed positive and negative feedback loops (Binzegger et al., 2004; Douglas and Martin, 2004; Bastos et al., 2012). At a macroscopic or systems level scale the organization of cortical connections seems to be hierarchical and modular, with dense excitatory and inhibitory connectivity within modules and sparse excitatory connectivity between modules (Hilgetag et al., 2000; Zhou et al., 2006; Meunier et al., 2010; Sadovsky and MacLean, 2013).

A number of studies considered effects of the structure of cortical connections on the existence of sustained cortical activity and on variability of the single-cell and population firing rates in that regime. Studies with random networks of sparsely connected excitatory and inhibitory neurons have shown that sustained irregular network activity can be produced when the recurrent inhibitory synapses are relatively stronger than the excitatory synapses (van Vreeswijk and Sompolinsky, 1996, 1998; Brunel, 2000; Vogels and Abbott, 2005; Kumar et al., 2008). Recently, the random network assumption has been relaxed and it has been shown that networks with clustered (Litwin-Kumar and Doiron, 2012), layered (Destexhe, 2009; Potjans and Diesmann, 2014), hierarchical and modular (Kaiser and Hilgetag, 2010; Wang et al., 2011; Garcia et al., 2012) connectivity patterns as well as with local and long-range connections plus excitatory synaptic dynamics (Stratton and Wiles, 2010) can generate cortical-like irregular activity patterns. Other works have focused on the role of signal transmission delays and noise in the generation of such states (Deco et al., 2009, 2010).

Emphasizing the role of the topological structure of the cortical networks, most of these models do not take into account the possible joint role of the multiple firing patterns of the different types of neurons that comprise the cortex. For example, descriptions in terms of the popular leaky integrate-and-fire model (see e.g., Vogels and Abbott, 2005; Wang et al., 2011; Litwin-Kumar and Doiron, 2012; Potjans and Diesmann, 2014), do not capture the diversity of firing patterns of cortical neurons (Izhikevich, 2004; Yamauchi et al., 2011). The exception is the model of Destexhe (2009), where complex intrinsic properties of the employed neurons correspond to electrophysiological measurements.

Intrinsic properties of cortical neurons like types of ion channels, and distributions of ionic conductance densities stand behind a variety of firing patterns. Based on their responses to intracellular current pulses, neurons with different patterns can be grouped into five main electrophysiological classes: regular spiking (RS), intrinsically bursting (IB), chattering (CH, also called fast repetitive bursting), fast spiking (FS) and neurons that produce low threshold spikes (LTS) (Connors et al., 1982; McCormick et al., 1985; Nowak et al., 2003; Contreras, 2004). Excitatory cells of the RS, IB, and CH classes are mostly pyramidal and glutamatergic, and comprise ~80% of cortical cells; their majority is of the RS type. On the other hand, inhibitory cells from the FS and LTS classes are of non-pyramidal shapes and GABAergic.

Given the variability of cortical firing patterns, the natural questions are: (i) how does the inclusion of neurons with varying intrinsic dynamics in a hierarchical and modular cortical network model affect the occurrence of SSA in the network? (ii) how does a combination of hierarchical and modular network topology with individual node dynamics influence the properties of the SSA patterns in the network?

To address these questions, we use a hierarchical and modular network model which combines excitatory and inhibitory neurons from the five cortical cell types. Higher complexity in comparison to previous models, in particular mixtures of different neuronal classes in non-random networks, hampers analytical studies. However, it is important to push modeling to these higher complexity situations that are closer to biological reality. Numerical simulations may give us insights on how to construct deeper analytical frameworks and shed light on the mechanisms underlying ongoing cortical activity at rest.

Our simulations show that SSA states with spiking characteristics similar to the ones observed experimentally can exist for regions of the parameter space of excitatory-inhibitory synaptic strengths in which the inhibitory strength exceeds the excitatory one. This is in agreement with the simulations of random networks made of leaky integrate-and-fire neurons mentioned above. However, our simulations disclose additional mechanisms that enhance SSA. The SSA lifetime increases with the number of modules, and when the network is made of LTS inhibitory neurons and a mixture of RS and CH excitatory neurons. These new mechanisms point to a synergy between network topology and neuronal composition in terms of neurons with specific intrinsic properties on the generation of SSA cortical states. The article is structured as follows: the next section specifies our neuron and network models and the measures used to characterize their properties; then, we describe our search in parameter space for regions which exhibit SSA and how the properties of these SSA depend on network characteristics. We end with a discussion of our main results and the possible mechanisms behind them.

## 2. Materials and methods

All functions, simulations, and protocols were implemented in C++. Ordinary differential equations were integrated by the fourth order Runge-Kutta method with step size of 0.01 ms. Processing of the results was performed in Matlab.

### 2.1. Neuron models

Neurons in our networks were described by the piecewise-continuous Izhikevich model (Izhikevich, 2003): the dynamics of the *i*-th neuron obeys two coupled differential equations,

(1) $$\begin{array}{l} {\dot{v}}_{i}=0.04v_{i}^{2}+5v_{i}+140-u_{i}+I_{i}(t)\\ {\dot{u}}_{i}=a(bv_{i}-u_{i}), \end{array}$$

with a firing condition: whenever the variable *v*(*t*) reaches from below the threshold value *v*_crit_ = 30 mV, the state is instantaneously reset, *v*(*t*) ↦ *c, u*(*t*) ↦ *u*(*t*) + *d*. The variable *v* represents the membrane potential of the neuron and *u* is the membrane recovery variable. Each resetting is interpreted as firing a single spike.

**Figure 1:**
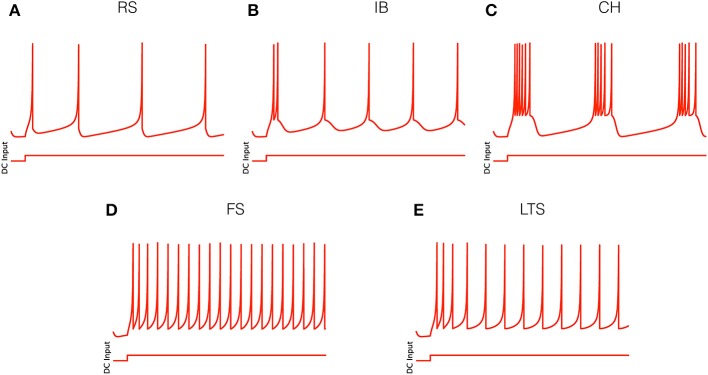
**Electrophysiological cell classes as modeled by Equation (1)**. Parameter values are given in the text. **(A)** Regular spiking (RS) neuron. **(B)** Intrinsically bursting (IB) neuron. **(C)** Chattering (CH) neuron. **(D)** Fast spiking (FS) neuron. **(E)** Low threshold spiking (LTS) neuron.

Appropriate combinations of the four parameters (*a, b, c, d*) generate the firing patterns of the five main electrophysiological cortical cell classes listed in the Introduction. We use the following sets of values (Izhikevich, 2003):

- for RS neurons: *a* = 0.02, *b* = 0.2, *c* = −65, *d* = 8 (Figure 1A);
- for IB neurons: *a* = 0.02, *b* = 0.2, *c* = −55, *d* = 4 (Figure 1B);
- for CH neurons: *a* = 0.02, *b* = 0.2, *c* = −50, *d* = 2 (Figure 1C);
- for FS neurons: *a* = 0.1, *b* = 0.2, *c* = −65, *d* = 2 (Figure 1D);
- for LTS neurons: *a* = 0.02, *b* = 0.25, *c* = −65, *d* = 2 (Figure 1E).

The term *I*_*i*_(*t*) in Equation (1) denotes the input received by neuron *i*. It can be of two types: external input and synaptic input from other neurons in the network. We modeled the latter as

(2) $$I_{syn,i}=\sum_{j\;\in \text{ presyn}}G_{ij}^{\text{ex/in}}(t)\left(E_{\text{ex/in}}-v_{i}\right),$$

where the sum extends over all neurons, presynaptic to neuron *i*, and *G*^ex/in^_*ij*_ is the conductance of the synapse from neuron *j* to neuron *i*, which can be either excitatory or inhibitory. The reversal potentials of the excitatory and inhibitory synapses are *E*_ex_ = 0 mV and *E*_in_ = −80 mV, respectively. We assume that the synaptic dynamics is event-driven without delays: when a presynaptic neuron fires, the corresponding synaptic conductance *G*^ex/in^_*ij*_ is instantaneously increased by a constant amount *g*_ex/in_. Otherwise, conductances obey the equation

(3) $$\frac{d}{dt}G_{ij}^{\text{ex/in}}(t)=-\frac{G_{ij}^{\text{ex/in}}(t)}{\tau_{\text{ex/in}}},$$

with synaptic time constants τ_ex_ = 5 ms and τ_in_ = 6 ms (Dayan and Abbott, 2001; Izhikevich and Edelman, 2008).

### 2.2. Network models

The hierarchical and modular architecture of our networks was constructed by a top-down method (Wang et al., 2011). In this approach, we started with a random network of *N* neurons connected with probability *p* and rewired it to obtain hierarchical and modular networks. Here we used two combinations of *N* and *p*: *N* = 512 with *p* = 0.02, and *N* = 1024 with *p* = 0.01. In both cases the ratio of excitatory to inhibitory neurons was 4:1. Excitatory neurons were purely of the RS type or a mixture of two types: RS (always present) with either CH or IB cells. Inhibitory cells were all of either FS or LTS type. A random network as the one described above constitutes one module and will be called here a network of hierarchical level *H* = 0. A network of hierarchical level *H* has 2^*H*^ modules (Wang et al., 2011), hence a network of hierarchical level *H* = 1 has 2 modules, a network with *H* = 2 has 4 modules, and so on.

Networks with *H* > 0 were generated by the following algorithm:

1. Randomly divide each module of the network into two modules of same size;
2. Each intermodular connection (*i* → *j*) is, with probability *R*, replaced by a new connection between *i* and *k* where *k* is a randomly chosen neuron from *the same module* as *i*. For inhibitory synapses we took *R* = 1: all intermodular inhibitory connections were deleted and only the local ones (intramodular) remained. In contrast, for excitatory connections, we took *R* = 0.9 which resulted in survival of a portion of those connections, and, thereby, in presence of both local and long-distance (i.e., intramodular and intermodular) excitatory links.
3. Recursively apply steps 1 and 2 to build networks of higher hierarchical levels.

**Figure 2:**
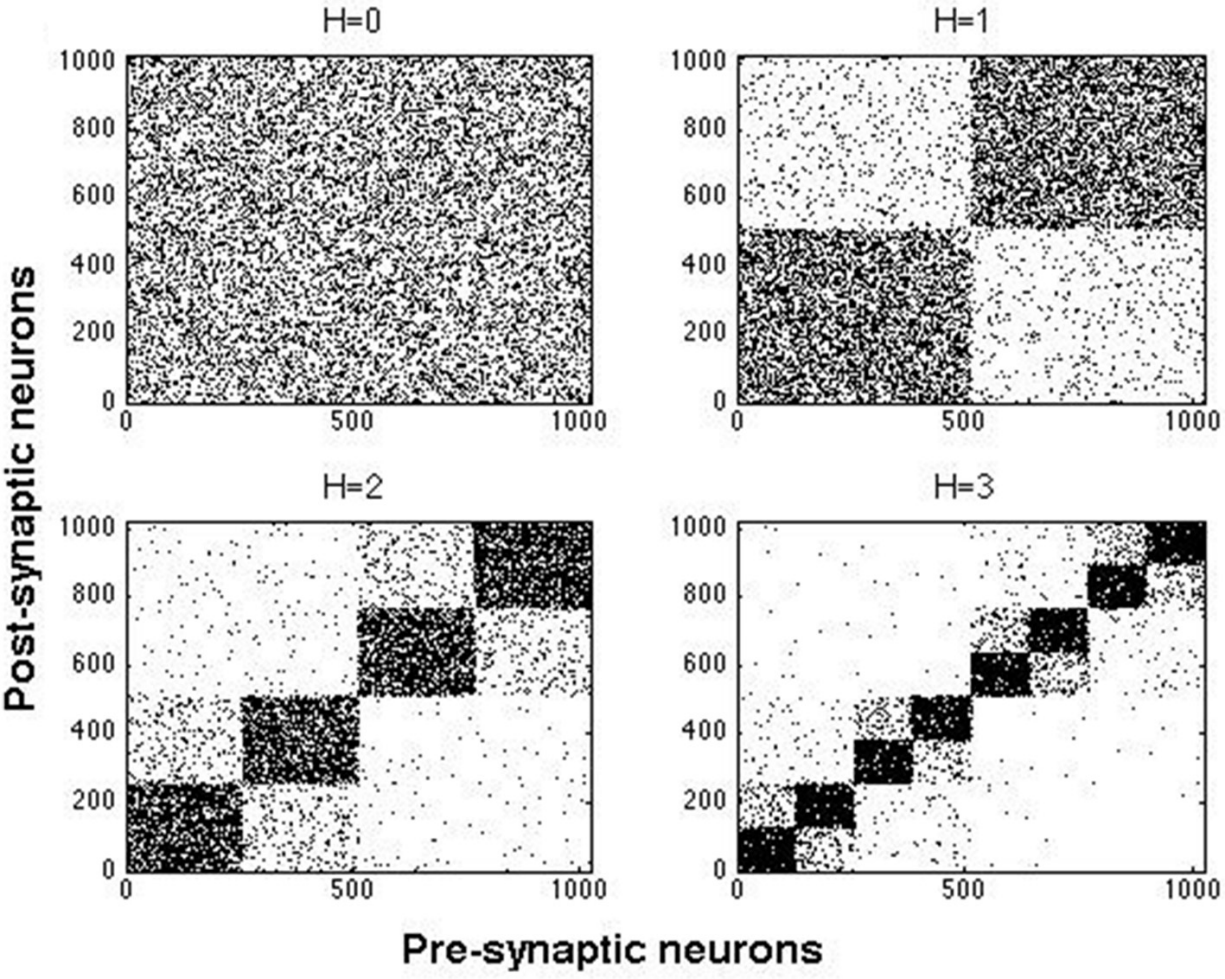
**Examples of connection matrices for hierarchical and modular networks at *H* = 0, …, 3 constructed with rebating probabilities given in text**. Each dot represents a connection from a presynaptic neuron to a postsynaptic one.

Figure 2 shows examples of hierarchical and modular networks constructed by the above procedure.

### 2.3. Network spiking characteristics

Here, we define the quantities and measures that characterize the spiking properties of single neurons and of the entire network.

The spike train of a neuron *i* is represented as (Gabbiani and Koch, 1998; Dayan and Abbott, 2001),

(4) $$x_{i}(t)=\sum_{t_{i}^{f}}\delta (t-t_{i}^{\;f}),$$

where {*t*^*f*^_*i*_} is the set of times at which a neuron *i* fires. The firing rate of this neuron over a time interval *T* is the number *n*_*i*_ of spikes which it fires during the interval, divided by *T*:

(5) $$f_{i}=\frac{n_{i}}{T}=\frac{1}{T}\int_{T}x_{i}(t^{\prime })dt^{\prime }.$$

Similarly, the mean firing rate of *N* neurons in the network over a time interval *T* is:

(6) $$\langle f\rangle =\frac{1}{N}\sum_{i\;=\;1}^{N}\frac{1}{T}\int_{T}x_{i}(t^{\prime })dt^{\prime }.$$

The time-dependent activity of the network *A*(*t*; Δ*t*) was defined as the total number of spikes fired by its neurons within a time interval Δ*t* around *t*:

(7) $$A(t;\Delta t)=\sum_{i\;=\;1}^{N}\int_{t}^{t\;+\;\Delta t}x_{i}(t^{\prime })dt^{\prime }.$$

Dividing it by the number of neurons, we obtain the time-dependent firing rate of the network:

(8) $$f(t;\Delta t)=\frac{1}{N}\sum_{i\;=\;1}^{N}\int_{t}^{t\;+\;\Delta t}x_{i}(t^{\prime })dt^{\prime }.$$

Equation (7) provides the variation of the number of active neurons in the network within the interval Δ*t* while Equation (8) gives the variation of the proportion of active neurons within Δ*t*. Since Δ*t* in both expressions will be fixed at 1 ms throughout this study, below we denote the time-dependent activity and firing rate of the network simply by *A*(*t*) and *f*(*t*).

Irregularity of network firing was characterized by two distributions: the distribution of interspike intervals (ISI) of all neurons in the network, and the distribution of the coefficients of variation (CV) of the ISIs of each neuron. The ISI distribution was formed by the set {ISI_*i*_}, *i* = 1, …, *N* for all neurons. To obtain the distribution of the CVs, we calculated for every neuron *i* the standard deviation σ_ISI_i__ of its ISI_*i*_ distribution normalized by the mean ISI_*i*_ for this neuron (Gabbiani and Koch, 1998):

(9) $$CV_{i}=\frac{\sigma_{{\text{ISI}}_{\text{i}}}}{{\bar{\text{ISI}}}_{i}},$$

and took the set of CV_*i*_ for all network neurons.

Basing on the values of these activity measures extracted from the raster plots of the simulations, we delineated the regions where SSA was observed on the plane of excitatory and inhibitory conductances (*g*_ex_, *g*_in_).

## 3. Results

### 3.1. Parameter dependence of SSA

Below, “architecture of the network” denotes the topology of the network, i.e., hierarchical level *H*, plus its composition, i.e., the types and proportions of participating neurons. A given network realization is then a network with fixed architecture, produced randomly by the algorithm from the preceding section.

We activated the network by injecting external current of amplitude *I*_stim_ into a proportion *P*_stim_ of the neurons for the time interval *T*_stim_. After stimulus termination, the network was left to evolve freely until the end of simulation time *T*_sim_. While this activation may look adequate enough from a physiological point of view, in the dynamical sense it plays only the role of setting initial conditions. In the course of stimulation, the system is driven to some position in the phase space, from where it is left to evolve on its own. The effect, of course, would be the same if the same starting state for free evolution was explicitly imposed from the beginning. However, external stimulation ensures that initial conditions are not just randomly chosen somewhere in the high-dimensional phase space, but lie close to typical pathways in its “physiologically reasonable” part. In the case of multistability (i.e., quiescent state and one or several kinds of SSA), variation of initial conditions can place the starting points in the attraction domains of different coexisting attractors.

#### 3.1.1. Parameter search

To gain insight into the properties of the system, we performed a preliminary study with small networks of 512 neurons and short simulation times *T*_sim_ = 350 ms in the parameter region of synaptic strengths *g*_ex_ ∈ [0, 1], *g*_in_ ∈ [0, 5], discretizing it with Δ*g*_ex_ = 0.1 and Δ*g*_in_ = 0.5. For each network realization and each parameter pair (*g*_ex_, *g*_in_) in this range, we took eight initial conditions in different regions of phase space. This was achieved by changing the proportion of stimulated neurons (either half of the neurons or all of them: *P*_stim_ = 1/2, 1), the amplitude of external current (*I*_stim_ = 20, 30) and the stimulation interval (*T*_stim_ = 80 ms, 120 ms).

**Figure 3:**
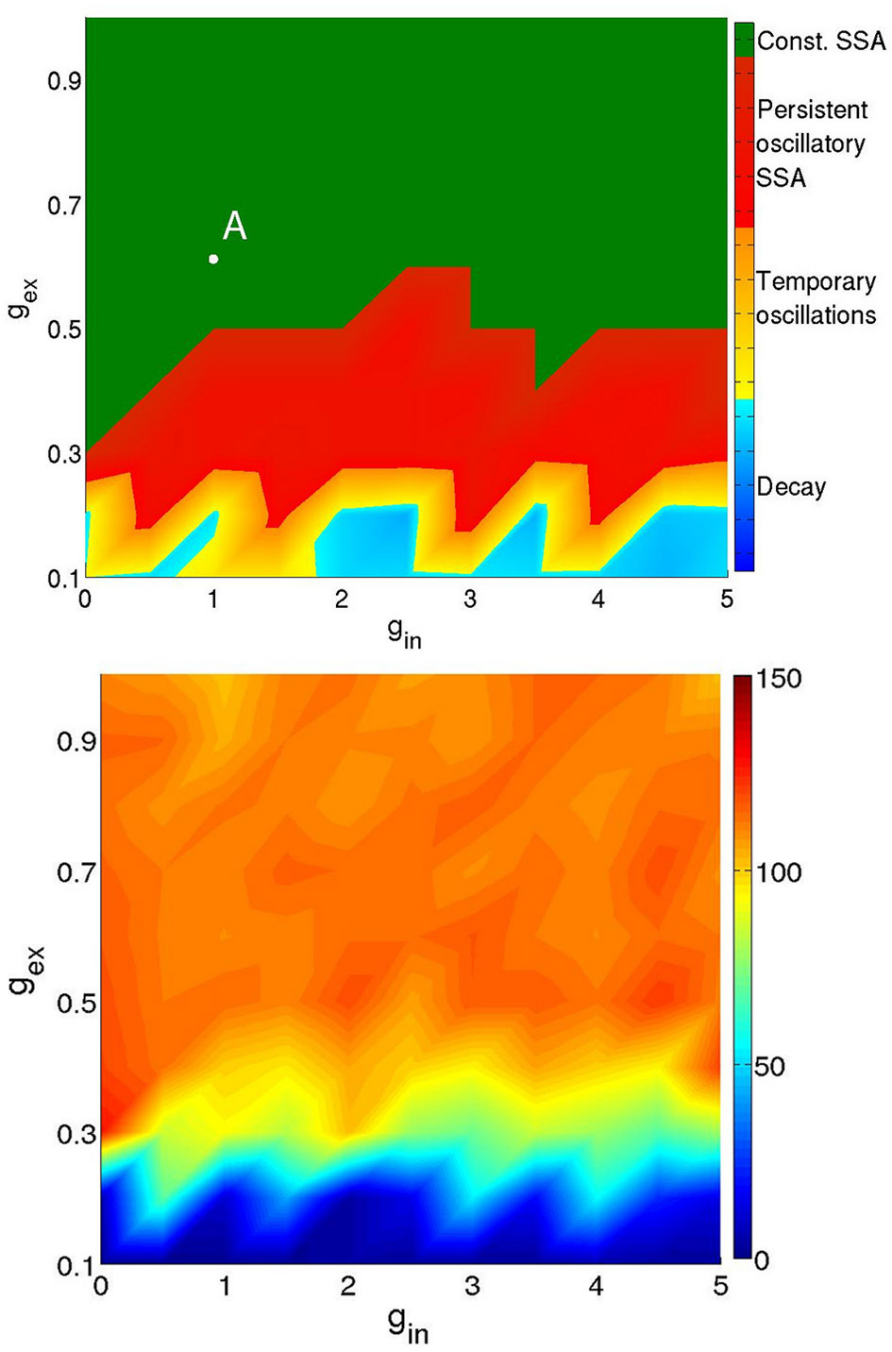
**Types of activity for a network of 512 neurons in 2 modules**. Neuronal types: 64% RS, 16%CH, 20% LTS. Activation parameters: *P*_stim_ = 1, *I*_stim_ = 20, *T*_stim_ = 80 ms. **Top:** duration of network activity. Green, constant SSA, red, persistent oscillatory SSA, yellow, temporary oscillatory SSA, blue, rapid decay. **Bottom:** Mean firing rate of the network during the active period. Firing rate ranges in Hz: see colorbox on the right.

Figure 3 presents a typical map of states under these conditions: the (*g*_ex_, *g*_in_)-diagram for a network of two modules (hierarchical level *H* = 1) where 20% of the excitatory neurons were of the CH class, all inhibitory neurons were of the LTS class, and the activation parameters were *P*_stim_ = 1, *I*_stim_ = 20, and *T*_stim_ = 80 ms.

**Figure 4:**
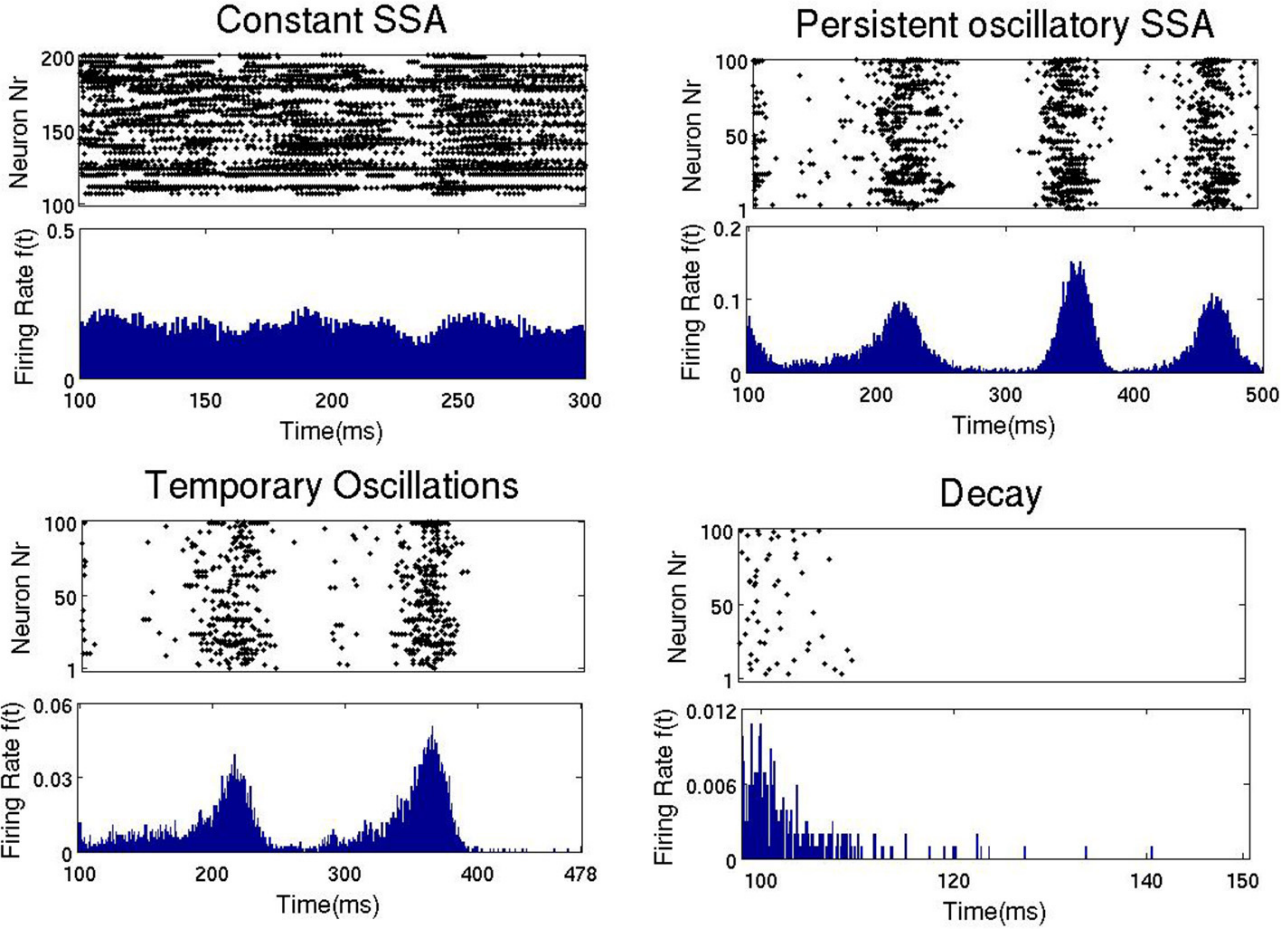
**Four types of network activity patterns**. Each panel shows the raster plot of the spiking activity for a sample of 100 network neurons **(Top)**, and the firing rate *f*(*t*) of all neurons **(Bottom)**. Constant SSA: point A in Figure 3 (*g*_ex_ = 0.6, *g*_in_ = 1). Persistent oscillatory SSA: point B in Figure 5 (*g*_ex_ = 0.12, *g*_in_ = 0.6). Temporary oscillations: point C in Figure 5 (*g*_ex_ = 0.09, *g*_in_ = 0.5). Decay: point D in Figure 5 (*g*_ex_ = 0.06, *g*_in_ = 0.2).

The top panel of Figure 3 shows the duration and type of network activity. The blue region corresponds to fast decay of activity after termination of the external input with network activity lasting not longer than 50 ms. We call this type of behavior “rapid decay.” The yellow region indicates large-scale network activity oscillations, when, for a certain time after activation, different groups of neurons fire synchronously, and decay afterwards. We call this behavior “temporary oscillatory activity.” The red region corresponds to the same type of network behavior as in the yellow one, but lasting until the end of the simulation, and we call it “persistent oscillatory SSA.” The green region indicates SSA with strongly irregular individual neuronal firing and more or less constant overall network activity; this behavior is referred to as “constant SSA.” Examples of these four behavioral patterns are visualized in Figure 4.

The bottom panel of Figure 3 represents the mean firing rate 〈*f*〉 of the neurons in the active period. The latter was defined as the time interval between the end of external stimulation and the time of the last spike in the network. If by the end of simulation neurons were still spiking, the whole duration of free evolution was taken as the length of active period. The regions corresponding to SSA yield somewhat unrealistic mean firing rates above 70 Hz in comparison with lower values found in cortex (Softky and Koch, 1993; Vogels and Abbott, 2005; Hromádka et al., 2008; Destexhe, 2009; Maimon and Assad, 2009; Haider et al., 2013). These high mean frequencies owe to CH and LTS neurons, which, in the green region of the diagram, can display firing rates as high as 600 Hz. In these regions, even the RS neurons can possess very high firing rates, in some cases as high as 200 Hz.

Regardless of those high firing rates, we studied the effects of changes in the network architecture, its realizations and initial conditions on the SSA. As a rough measure of the latter, we regarded the area occupied by the SSA regions on the parameter plane of (*g*_ex_, *g*_in_). For this small network, we summarize our observations as follows:

- Increase of the hierarchical level *H* (i.e., the number of network modules) under fixed other conditions led to growth of the SSA area;
- If the second excitatory neuron type (besides the RS neurons) was CH, increase of its proportion led to growth of the SSA area;
- If the second excitatory neuron type was IB, variation of its proportion displayed no clear influence on the SSA area;
- Under fixed other characteristics, replacement of FS inhibitory neurons by LTS inhibitory neurons increased the SSA area.

We did not observe noticeable changes in the SSA area for different network realizations and/or activation parameters. The few observed changes were mostly seen as small displacements along the border between the red and yellow regions in the top diagram of Figure 3 (data not shown). These changes became significant in the lower left part of the diagram (data also not shown), where the mean firing rates were closer to biological values. Therefore, below we concentrate on this parameter region, which we call the region of low synaptic strengths.

### 3.2. SSA for low synaptic strengths

From now on we consider a larger network consisting of 1024 neurons within the parameter range of weaker synaptic strengths: *g*_ex_ ∈ [0.05, 0.15], *g*_in_ ∈ [0, 1].

**Figure 5:**
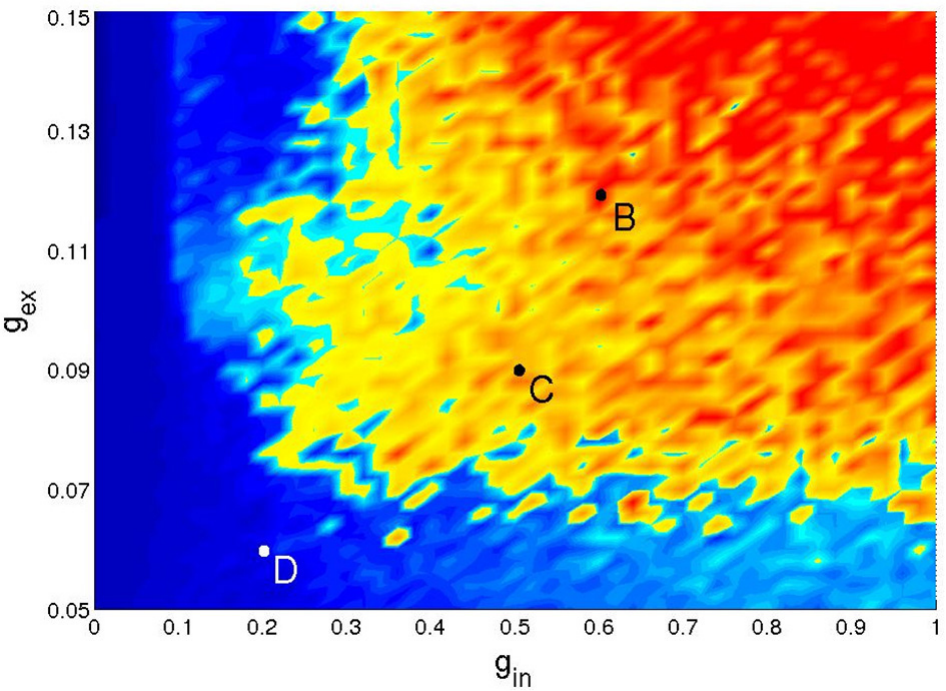
**Network activity on the parameter plane of low synaptic strengths: a typical distribution of network activity patterns for 2^10^ neurons**. Network parameters and the coloring scheme as in the top panel of Figure 3.

Figure 5 gives an example of the (*g*_ex_, *g*_in_) diagram for low synaptic strengths (discretized on a 50 × 50 grid with Δ*g*_ex_ = 0.002 and Δ*g*_in_ = 0.02). It corresponds to a network with hierarchical level *H* = 1, 20% of its excitatory neurons of the CH type, inhibitory neurons of the LTS type, and the following activation parameters: *P*_stim_ = 1/2, 10 ≤ *I*_stim_ < 20 and *T*_stim_ = 100 ms. The simulation was prolonged up to 1000 ms. The lifetime of activity strongly depends on the initial conditions: for a given network realization, some initial conditions would result in SSA while others would not. Therefore, only a statistical characterization of activity makes sense. In each point of the parameter grid we chose 10 different initial conditions, followed the evolution and plotted the maximal lifetime. The resulting diagram captures the generic properties of all studied network architectures in the region of low synaptic strengths: in all cases no constant SSA was detected, and self-sustained activity, if present, was oscillatory. The striking feature is the highly fragmented shape of the SSA region which is located in the upper right corner of the diagram. Changing the activation protocol, under the fixed network architecture, we observed similar fragmented structures with slightly different configurations (not shown). For neighboring initial conditions, prepared by varying the stimulation time within several integration steps, the lifetime of network activity varied over the range from few milliseconds up to 10^4^ ms. Notably, even at low values *g*_ex_ (the bottom part of the diagram) there is some probability to observe SSA with three or four subsequent epochs of high synchronous activity.

High sensitivity with respect to initial conditions is a hallmark of dynamical chaos. On the other hand, at least within the range of low synaptic strengths, the chaotic regime is hardly an attractor, since activity typically dies out after a long or short transient: trajectories end up at the trivial stable state where all neurons are at their resting potential. Systems which, for typical initial conditions, exhibit chaos up to a certain time and then, often abruptly, switch to non-chaotic dynamics, are known as transiently chaotic (Lai and Tél, 2011). Detailed investigation of chaotic sets in this high-dimensional system is out of the scope of our present study and will be reported elsewhere.

Based on our observations, we may say with a high certainty that the SSA states in the domain of low synaptic strengths are due to transient chaos and therefore have finite lifetimes. Increasing the synaptic strengths to higher parameter values, e.g., (*g*_ex_ ~ 1, *g*_in_ ≳ 2) may lead to a situation where the transient chaotic set turns into an attractor and the SSA becomes incessant. However, as remarked above, this would result in very high firing frequencies and, hence, would hardly correspond to biologically realistic cases.

The fact that we are dealing with transient SSA makes the analysis somewhat ambiguous: there seems to be no definite way to draw a sharp boundary in the parameter space, between the domains with SSA and those without it. However, under each fixed set of parameters, we can evaluate the probability of having SSA with a given duration. This, of course, requires statistics for a sufficient number of initial conditions.

First, we partitioned the (*g*_ex_, *g*_in_) diagram of low synaptic strengths into sixteen distinct domains. For all network architectures and each of the domains we tested 120 different initial conditions, prepared by external stimulation: we varied the proportion of stimulated neurons *P*_stim_ = 1, 1/2, 1/8, 1/16, the input current *I*_stim_ = 10, 20 and the stimulation time *T*_stim_ = 50, 52, …, 78 ms. In this way we intended to lead the system to distinct regions of the phase space (presumably governed by the number of stimulated neurons), and then, by varying *T*_stim_, to gather statistics within these regions. Each run ended when the activity died out completely, or else at 10^4^ ms.

**Figure 6:**
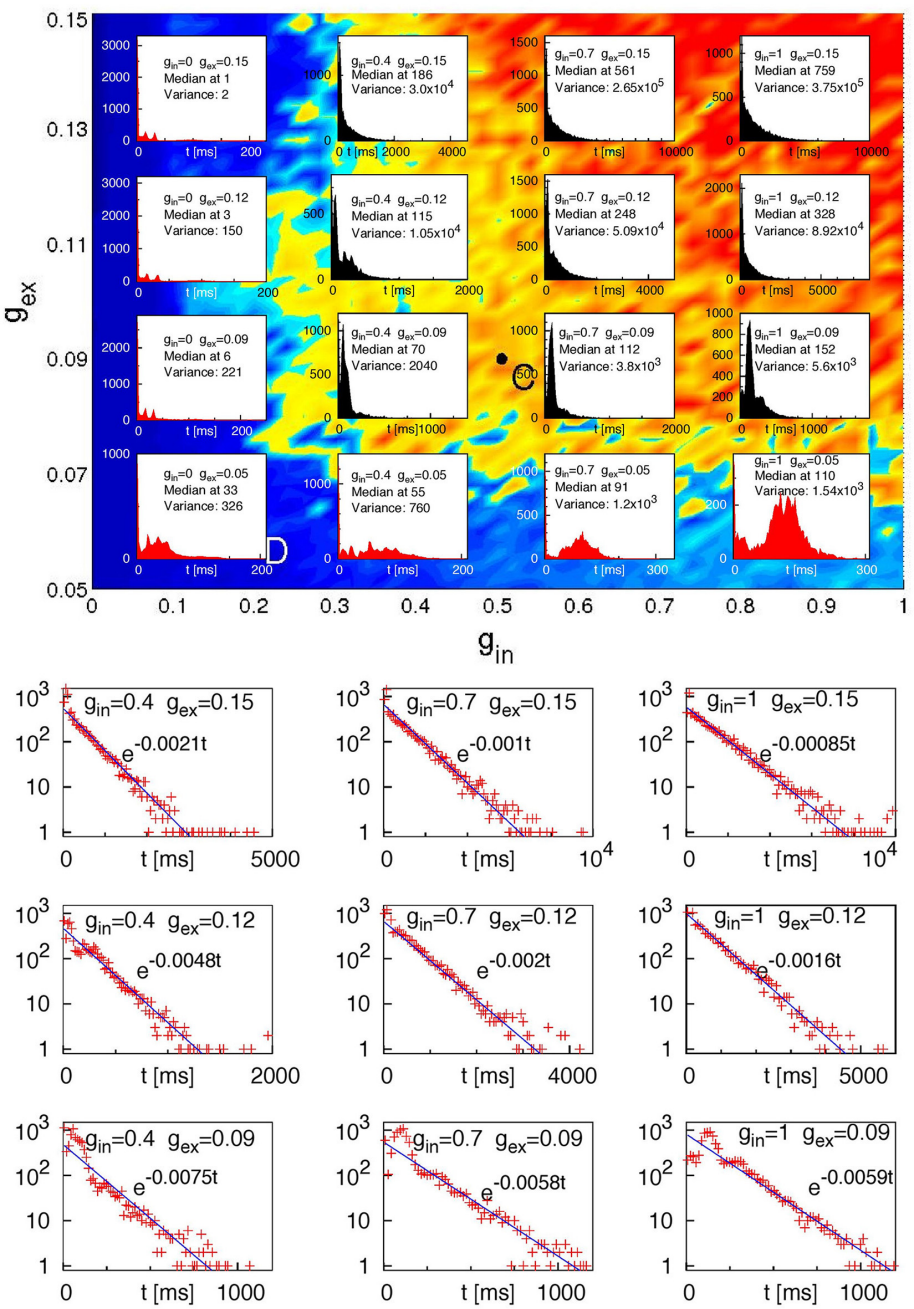
**Lifetime distributions for a network of 2^10^ neurons with four modules (*H* = 2); 20% of the excitatory neurons are CH; the inhibitory neurons are LTS. Top:** Histograms of lifetimes, with medians and variances, for 10^4^ different initial conditions at sixteen pairs (*g*_ex_, *g*_in_). **Bottom:** ordinate values on the logarithmic scale for 9 upper right (“black”) histograms from the top panel. Straight lines: fitted exponential dependencies.

We observed that regardless of the network architecture in the absence of inhibition (*g*_in_ = 0) or at very low excitatory synaptic strength (*g*_ex_ = 0.05) no cases of SSA occurred and the system relaxed toward the fixed point in a non-chaotic way for all 120 tested initial conditions. Figure 6 displays extended statistics for a network with four modules (*H* = 2) where 20% of the excitatory neurons are CH, and the inhibitory neurons are LTS. For each of the sixteen (*g*_ex_, *g*_in_) pairs, over a thousand different initial conditions were used. The top panel shows the corresponding lifetime distributions. At sufficiently high inhibition and excitation, for most of the network architectures these distributions display exponential decay. Replotting on the logarithmic scale the ordinate for the nine cases in the upper right corner of the top panel (the bottom panel of Figure 6) confirms this observation: the probability of finding a chaotic transient SSA with lifetime τ decays exponentially in τ, at a rate which depends on the network parameters. Such exponential distributions of the lifetime of chaotic transients are typical for systems with transient chaotic behavior (Lai and Tél, 2011).

Concentrating on the four pairs (*g*_ex_, *g*_in_) from the far upper right corner in Figure 6 (*g*_ex_ = 0.12, 0.15, and *g*_in_ = 0.7, 1) which showed most cases of transient SSA, we performed additional simulations for all architectures, creating in each case a few thousands initial conditions by varying the stimulation time in the range of 50 ms to 175 ms and/or the amplitude of the stimulus in the range of 10–30 and/or the proportion of stimulated neurons *P*_stim_ = 1, 1/2, 1/8, 1/16.

In the next subsection we present the obtained results and demonstrate that dependence of SSA on the values of *g*_ex_ and *g*_in_ varies strongly in response to changes in the network architecture.

### 3.3. Changes with respect to network architecture

Here, we describe the changes in the SSA states caused by variation of the network architecture in the region of low synaptic strengths. Below, we basically refer to the four investigated pairs (*g*_ex_, *g*_in_) corresponding to the most active domain of the parameter plane, since there the changes are better visible, and the tendencies can be better inferred from the statistics based on few thousands initial conditions for each of the parameter pairs and each of the network architectures. Results based on the statistics gathered for the 120 initial conditions for the neighboring regions display similar tendencies but are less distinct.

The findings are summarized in Table 1. There, we used as observable the value of the median for the distribution of the lifetimes of SSA. Being interested only in SSA cases, we excluded all trials which resulted in rapid decay or very short oscillatory activity: only the datasets for which, after the end of the stimulation, the lifetime exceeded 300 ms, were processed. From a dynamical point of view this corresponded to a choice of trajectories that for a certain time lived on the chaotic set. Remarkably, this cut off of the short-lived trajectories led to a drastic reduction of the number of trials in the analyzed distributions. Especially in the case of architectures and synaptic parameters under which the probability of long-lived SSA was low, this increased the influence of statistical outliers on the calculated values. Therefore, in the following we can only speak about tendencies. A systematic quantitative research would require a huge amount of trials, beyond our current computational capabilities. We point out that altogether over 10^6^ initial conditions were simulated and analyzed.

**Table 1:** **Effect of the network architecture on SSA for four different pairs of synaptic strengths *g*_ex_ and *g*_in_**.

**Medians [ms]**											
**Excitatory neurons**		**Inhibitory neurons: LTS**				**Excitatory neurons**		**Inhibitory neurons: FS**			
		**(*g*_ex_, *g*_in_)**						**(*g*_ex_, *g*_in_)**			
	**H**	**(0.12, 0.7)**	**(0.15, 0.7)**	**(0.12, 1)**	**(0.15, 1)**		**H**	**(0.12, 0.7)**	**(0.15, 0.7)**	**(0.12, 1)**	**(0.15, 1)**
RS	0	408	365	544	431	RS	0	xxx	xxx	xxx	xxx
	1	506	428	707	535		1	346	329	372	357
	2	603	674	850	834		2	423	519	490	554
20%CH	0	372	500	421	573	20%CH	0	xxx	375	355	368
	1	449	583	519	653		1	343	363	356	370
	2	618	1011	756	1209		2	441	521	475	555
40%CH	0	729	2343	759	2258	40%CH	0	397	663	396	565
	1	1027	3821	1086	3566		1	379	434	379	439
	2	2866	9907	4344	9907		2	1036	1735	1210	1734
20%IB	0	339	359	368	374	20%IB	0	xxx	xxx	xxx	xxx
	1	385	360	435	385		1	xxx	xxx	337	333
	2	474	527	582	607		2	403	457	430	490
40%IB	0	317	379	330	380	40%IB	0	xxx	xxx	xxx	xxx
	1	360	360	376	364		1	xxx	xxx	335	327
	2	417	557	484	632		2	370	442	409	471

*Medians of activity lifetimes are restricted to cases of SSA exceeding 300 ms after the end of stimulation. “xxx” denotes networks for which such cases did not occur or were very seldom*.

We start the analysis with networks where all excitatory neurons are RS, whereas inhibitory neurons are either LTS or FS (see rows in Table 1 corresponding to RS neurons). In this range of synaptic strengths and for hierarchical level *H* = 0 the combination RS-FS could hardly lead to SSA: the activity was seldom longer than 100 ms, and was followed by direct decay to the stable state. In contrast, the RS-LTS combination delivered cases of SSA. Albeit relatively rare (recall the exponential distribution in Figure 6), for the RS-LTS network some SSA states could display lifetimes longer than 1000 ms. Changing the number of modules had little effect on SSA duration for RS-FS networks due to low probability of finding SSA in this case (see above). Nevertheless, in the network with four modules (*H* = 2) we observed many cases of SSA with lifetimes longer than 500 ms, while none was observed for a random network with *H* = 0. For RS-LTS networks the effect of increase in the number of modules was more articulate: The longest lifetimes of the SSA grew from a few hundred ms for random networks (*H* = 0) to a few thousand ms for modular networks (*H* = 1, 2).

Introduction of CH neurons as a second type of excitatory neurons led to a noticeable increase in the lifetime expectancy of SSA for the *H* = 0 case, both for LTS and FS inhibitory neurons. In the former case, the increase was more pronounced. For the case of LTS inhibitory neurons, the presence of just 20% of CH neurons in the excitatory population slightly expanded the SSA domain of synaptic conductances toward lower values of the (*g*_ex_, *g*_in_) diagram (not shown). Besides this, in the upper right part of the diagram (see rows in Table 1 corresponding to LTS cases with *H* = 0 and 20% or 40%CH) the probability to get a durable (over 1000 ms) SSA became higher. Increase of the percentage of CH neurons to 40% confirmed the tendency of growing SSA lifetime expectancy in the middle part of the (*g*_ex_, *g*_in_) diagram (not shown). Remarkably, in the upper right region of the diagram the distribution was no longer exponential, at least not in the examined range of lifetimes. The median of the lifetime distribution became significantly higher (above 2000 ms at *g*_ex_ = 0.15), and at high modularity it became more probable to get SSA with duration up to 10^4^ ms (which means over 100 subsequent epochs of collective activity) than not to observe SSA at all. In the case of networks with FS inhibitory neurons, the presence of CH neurons as the second type of excitatory neuron had a similar effect of increasing the SSA lifetime expectancy, but by far not so strong. In fact, for the middle part of the diagram the effect was barely noticeable, even when the proportion of CH neurons was 40% (not shown), and it hardly makes sense to speak of SSA in this case. In the upper right corner of the diagram (see rows in Table 1 corresponding to FS cases with *H* = 0 and 20%CH or 40%CH), cases of SSA were detected but the respective lifetime medians indicate that lifetimes longer than a few 100 ms are seldom. At higher modularity levels the effect of CH neurons as a second type of excitatory neurons became more visible. In the configuration with RS and CH excitatory neurons and LTS inhibitory neurons, hierarchical levels *H* = 1, 2 allowed the SSA lifetime to reach values ~10^4^ ms in the upper right corner of the diagram (see rows in Table 1 corresponding to LTS cases with *H* = 1, 2 and 20% or 40%CH) and a few thousand ms in the middle part of the diagram (not shown). The same tendency, but with a weaker effect, was observed when the inhibitory neurons belonged to the FS class (see Table 1 rows corresponding to FS cases with *H* = 1, 2 and 20% or 40%CH): here at *H* = 2 and with 40% of CH neurons the distributions of activity lifetimes had medians that exceeded 1000 ms and some initial conditions resulted in SSA states with lifetimes ~10^4^ ms.

At *H* = 0, the effect of IB neurons as a second type of excitatory neuron, compared to purely RS excitatory neurons, was relatively weak, especially when the inhibitory neurons were of the FS class since in that case SSA was almost absent (see Table 1 rows corresponding to FS cases with *H* = 0 and 20% or 40%IB). This is not surprising, since the difference between RS and IB neurons is not so strong as the difference between RS and CH neurons, especially in presence of irregularity of synaptic currents in the network. The effect was modest for LTS inhibitory neurons as well. However, noticeably and, somewhat surprisingly, this case displayed a clear *negative* tendency on the SSA lifetime (see Table 1 rows corresponding to LTS cases with *H* = 0 and 20% or 40%IB). In all configurations with IB neurons, growth of the number of modules resulted in the increase of the SSA lifetime (see rows in Table 1 corresponding to *H* = 1, 2 and 20% or 40%IB).

Our calculations unambiguously confirmed that modularity of the network favored SSA and extended its average lifetime (compare in Table 1 rows for *H* = 0 with rows for *H* = 1, 2). This effect is well seen e.g., at *g*_ex_ = 0.12, *g*_in_ = 0.7 in an exemplary network of 1024 neurons in which the inhibitory neurons are of the LTS type, and the CH neurons make 20% of the excitatory ones. At these parameter values (cf. the bottom panel of Figure 6) the probability to find an SSA with duration τ decays as exp (−ατ). For *H* = 0, 1, 2 the fitted values of α were, respectively, 7.47 × 10^−3^, 3.74 × 10^−3^, and 1.74 × 10^−3^ ms^−1^: each modularity level approximately doubles the expectancy of SSA duration.

### 3.4. Quantitative characteristics

Below we present characteristics of spiking dynamics in the studied networks: activities, frequency spectra, firing rates, interspike intervals and coefficients of variation (see Section 2.3), both globally and for different subpopulations of neurons.

We start with computation of these measures for several initial conditions in a network with fixed architecture and values of (*g*_ex_, *g*_in_) which ensure sufficiently long SSA.

**Figure 7:**
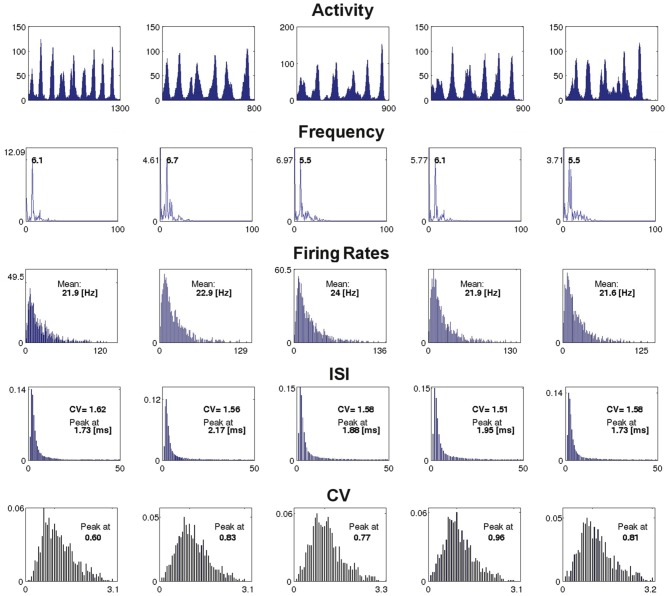
**Example of dependence of the spiking properties on the initial conditions**. The figure shows the network measures for a fixed network architecture: *H* = 2, RS excitatory neurons, LTS inhibitory neurons, *g*_ex_ = 0.15, *g*_in_ = 0.7, and five different initial conditions, one for each column. The first row: network activity *A*(*t*) over the active period, from the end of the external stimulation (time 0 in the horizontal axis) until last spike of a network (indicated by the number under the right end of the time axis, in ms). The second row: global frequency spectrum of the activity (horizontal axis: frequency in Hz, vertical axis: amplitude). The third row: distribution of the firing rates over the ensemble of neurons in the active period (the mean of each distribution is shown inside the corresponding plot and the maximal rate is shown at the extreme right of the horizontal axis). The fourth row: distribution of the ISIs (in ms) over the ensemble of neurons for the active period (with CV and the peak value of the distribution indicated inside each plot). The fifth row: distribution of the CVs of the ISIs of the network neurons; the peak of each distribution is shown inside the plot.

Figure 7 presents characteristics for an example network of four modules (*H* = 2), with RS excitatory neurons and LTS inhibitory neurons at *g*_ex_ = 0.15, *g*_in_ = 0.7, computed between the end of the external input and the last network spike. For all runs the duration of SSA exceeded 500 ms. Each column of the figure stands for a different set of initial conditions, whose SSA lifetime is shown in the activity plots on the first row. In all cases the type of activity pattern is oscillatory SSA (the only observed SSA type at low synaptic strengths). Further rows in the figure show the global frequency distribution of the network activity calculated via the Fourier transform, distributions of the neuronal firing rates *f*_*i*_, of the interspike intervals (ISI) with their coefficients of variation (CV) and, in the last row, of the CVs for the ISIs of individual neurons.

The measures presented in Figure 7 disclose little reaction to variation of initial conditions; in general, this observation holds for networks with other kinds of architecture as well. In several examples, especially for higher hierarchical levels, variability was more pronounced; this referred to amplitudes of the leading frequencies in the spectra (whereby the frequencies themselves stayed nearly constant), and can be attributed to non-coincidence of durations of oscillatory epochs in different modules. Notably, in all studied network architectures at all combinations of synaptic strengths we found no indicator that would signalize the approaching abrupt cessation of the SSA: from the point of view of average characteristics of activity, there is no visible difference between the short and the durable SSA.

Weak sensitivity of the SSA characteristics with respect to initial conditions supports our assumption that the state of SSA corresponds to wandering of all trajectories in the phase space over the same chaotic set which possesses well defined statistical characteristics but is (at least, in the domain of weak synaptic strengths) not an ultimate attractor of the system. Within the high-dimensional phase space of the network, this set appears to lie in a kind of relatively low-dimensional “channel”; nearby trajectories are quickly attracted by this channel, move along it for a certain time, and finally escape to the equilibrium.

Regarding the type of spiking behavior, the measures shown in Figure 7 reveal an irregular network activity. The distribution of the neuronal firing rates, clearly non-Gaussian, is asymmetric and long-tailed. The ISI distribution, non-Gaussian as well, is close to exponential, as can be expected for nearly Poissonian behavior. The distribution of the CVs of the ISIs is broad and asymmetric with average value ≳1. We recovered these features in all encountered SSA states in the region of low synaptic strengths.

Given this point, we proceed to the description of how different network compositions affect the activity characteristics. The general results on the effect of network architecture are summarized in Table 2 for excitatory neurons and Table 3 for inhibitory neurons. In these tables, each of the activity characteristics is calculated from the average over 10 different initial conditions resulting in SSA with lifetimes above 700 ms.

**Table 2:** **Effect of the network architecture on characteristic measures of the excitatory neurons at synaptic strengths *g*_ex_ = 0.15, *g*_in_ = 1**.

**Characteristic measures for excitatory neurons**									
**Excitatory neurons**	**H**	**LTS inhibitory neurons**				**FS inhibitory neurons**			
		**Firing rate median**		**ISI CV**		**Firing rate median**		**ISI CV**	
		**RS**	**CH/IB**	**RS**	**CH/IB**	**RS**	**CH/IB**	**RS**	**CH/IB**
RS	0	15	–	1.2	–	xxx	–	xxx	–
	1	14	–	1.2	–	15	–	1.2	–
	2	13	–	1.4	–	13	–	1.5	–
20%CH	0	31	79	1.9	3.2	29	63	2.0	3.2
	1	30	79	1.8	3.0	26	64	2.0	3.1
	2	26	69	1.9	3.0	22	56	2.0	3.2
40%CH	0	48	124	2.2	3.3	40	94	2.5	4.0
	1	46	122	2.2	3.3	34	82	2.4	3.7
	2	43	114	2.1	3.3	31	84	2.6	4.1
20%IB	0	22	35	1.7	2.3	xxx	xxx	xxx	xxx
	1	19	28	1.5	2.0	xxx	xxx	xxx	xxx
	2	16	28	1.7	2.2	16	27	1.7	2.2
40%IB	0	26	41	2.1	2.7	xxx	xxx	xxx	xxx
	1	24	38	1.9	2.5	xxx	xxx	xxx	xxx
	2	21	36	2.0	2.5	19	33	2.0	2.5

*Measures are computed from average over 10 different trials with lifetimes of the SSA over 700 ms. “xxx” denotes networks in which such lifetimes were observed in less than 10 trials*.

**Table 3:** **Effect of the network architecture on characteristic measures of the inhibitory neurons at synaptic strengths *g*_ex_ = 0.15, *g*_in_ = 1**.

**Characteristic measures for inhibitory neurons**									
**Excitatory neurons**	**H**	**Inhibitory neurons: LTS**							
		**Total**		**Firing rate**			**ISI**		**CV peak**
		**Excitation**	**Inhibition**	**Mean**	**Median**	**Max**	**Peak**	**CV**	
RS	0	0.015	0.037	38	32	121	1.7	1.7	1.2
	1	0.015	0.039	39	32	129	1.9	1.6	1.2
	2	0.016	0.040	40	33	119	1.7	1.7	1.1
20%CH	0	0.046	0.076	76	59	268	1.2	2.4	1.5
	1	0.044	0.077	77	61	264	1.2	2.4	1.6
	2	0.044	0.077	77	66	246	1.3	2.3	1.7
40%CH	0	0.093	0.123	123	98	367	1.2	2.7	1.8
	1	0.087	0.123	123	104	384	1.2	2.7	2.0
	2	0.085	0.118	118	99	346	1.2	2.7	2.0
20%IB	0	0.025	0.050	50	37	179	1.1	2.2	1.3
	1	0.023	0.049	49	38	170	1.2	2.1	1.3
	2	0.025	0.051	51	40	171	1.2	2.1	1.1
40%IB	0	0.036	0.061	61	43	208	1.0	2.6	1.7
	1	0.033	0.060	60	44	216	1.0	2.5	1.6
	2	0.035	0.064	64	50	212	1.1	2.3	1.5
**Excitatory neurons**	**H**	**Inhibitory neurons: FS**							
		**Total**		**Firing rate**			**ISI**		**CV peak**
		**Excitation**	**Inhibition**	**Mean**	**Median**	**Max**	**Peak**	**CV**	
RS	0	xxx	xxx	xxx	xxx	xxx	xxx	xxx	xxx
	1	0.017	0.043	43	30	181	1.4	1.9	1.4
	2	0.018	0.042	42	30	150	1.2	2.2	1.0
20%CH	0	0.047	0.085	85	51	368	1.1	2.9	2.2
	1	0.043	0.083	83	49	350	1.1	2.9	1.7
	2	0.041	0.080	80	53	315	1.0	3.1	1.5
40%CH	0	0.079	0.127	127	79	491	0.9	3.9	1.8
	1	0.074	0.128	128	66	493	1.0	3.8	2.2
	2	0.072	0.125	125	75	471	0.8	4.4	1.9
20%IB	0	xxx	xxx	xxx	xxx	xxx	xxx	xxx	xxx
	1	xxx	xxx	xxx	xxx	xxx	xxx	xxx	xxx
	2	0.026	0.054	54	35	227	1.0	2.6	1.2
40%IB	0	xxx	xxx	xxx	xxx	xxx	xxx	xxx	xxx
	1	xxx	xxx	xxx	xxx	xxx	xxx	xxx	xxx
	2	0.035	0.068	68	43	279	0.9	2.9	1.3

*Measures are computed from average over 10 different trials with lifetimes of the SSA over 700 ms. “xxx” denotes networks in which such lifetimes were observed in less than 10 trials*.

For networks with excitatory neurons of RS type only, comparisons between the cases with LTS and FS inhibitory neurons for fixed synaptic strengths and various initial conditions showed no significant difference in the mean firing rates of the excitatory neurons (see in Table 2 rows for RS cases). Introduction of CH neurons as the second type of excitatory neuron led to a significant increase in the firing rate of excitatory RS neurons (see Table 2 rows for 20% or 40%CH). In networks with LTS inhibitory neurons, when the CH neurons comprised 20% of all excitatory neurons the median firing rate of RS neurons doubled and when the proportion of CH reached 40% the median firing rate of RS neurons tripled. In networks with FS inhibitory neurons these increments in RS neurons firing rate were less pronounced, the growth factors being approximately 1.7 (20%CH) and 2.3 (40%CH). On the other hand, the effect of IB neurons was much weaker and (based on the few relevant data for FS inhibitory neurons) independent of the type of inhibitory neuron (see Table 2 rows corresponding to 20% or 40%IB). Remarkably, the effect of modularity on the firing rate of excitatory neurons was not very pronounced (see Table 2), and median firing rates for *H* > 0 levels remained in the same range as in the case of a random network topology (*H* = 0).

In the preceding subsection we noted that presence or absence of particular types of neurons strongly influences the probability of SSA. Intuitively, this could be expected, due to the different amounts of excitation and inhibition they provide to the network, an effect also known for leaky integrate-and-fire neurons (Brunel, 2000; Kumar et al., 2008). However, if this were the only reason, the lifetime distributions for networks with LTS inhibitory neurons should be similar to those for FS neurons at lower inhibitory synaptic strength, which was not confirmed by numerics (see Table 1).

Effect of the type of inhibitory neuron on the amounts of excitation and inhibition produced by the network is shown in Table 3. The first two columns of Table 3 (for LTS and FS neurons respectively) represent the total excitation and the total inhibition produced by the network, measured respectively as the total number of spikes produced by excitatory and inhibitory neurons normalized over the activity period. The other columns represent the activity measures for networks with LTS or FS neurons as introduced above. Remarkably, the exchange of LTS and FS neurons at fixed modularity level and percentage of the second type of excitatory neurons did not have a significant effect on the total excitation produced by the network. This can be seen in a comparison of the first column in Table 3 for LTS or FS neurons respectively. However, the maximal firing rates (and hence, quite often, the corresponding mean values) of the FS neurons were consistently higher than for the LTS neurons (see columns for maximum and mean firing rates in Table 3). At the same time many FS neurons displayed very low firing rates, which resulted in lower medians of the distributions for FS neurons than for LTS neurons (see columns for median firing rates in Table 3). This tendency was preserved not only when all excitatory neurons were RS but also in the cases with a second type of excitatory neurons and also for different modularity levels (see Table 3).

These characteristics suggest that the firing rate distribution of LTS neurons is more uniform, both in space and time, than the firing rate distribution of FS neurons. This is not indeed surprising: As the name suggests, a LTS neuron needs less excitatory input in order to reach a spiking threshold (~2.8 mV) in comparison to a FS neuron (~3.4 mV). On the other hand, once the threshold is reached, a FS neuron spikes much more often (at a frequency ~140 Hz for an input of *I* = 10) compared to the LTS neuron (~80 Hz for the same input). Therefore, when embedded in a network, the LTS neurons require less correlated excitatory input in order to spike, which makes them more sensitive. The FS neurons, in contrast, respond only to relatively high correlated excitation, hence their population includes many non-active neurons along with few ones with very high spiking rates. As a consequence, while the total inhibition produced by the network is comparable for both types of inhibitory neurons (see the second column in Table 3 for LTS or FS neurons respectively), the inhibitory spreading in the case of networks with FS neurons is less efficient than in networks with LTS neurons, being concentrated on the few relevant postsynaptic neurons. The end result is that networks built of LTS cells possess more inhibitory neurons with moderate spiking frequencies than networks built of FS cells.

Presence (both of 20% or 40%) of CH neurons in the network did not affect the tendency described above in different behavior of the two types of inhibitory neurons: the mean firing rate and the corresponding maximal firing rate of the FS neurons was higher than for the LTS neurons; however, the median of the firing rate distribution was still lower for FS neurons than for LTS neurons (see Table 3). This again meant presence of a few very active FS inhibitory neurons on one side of the distribution and of many weakly active FS neurons on its other side. In comparison, most of the LTS neurons were active with moderate firing rates.

Further, we considered the firing rates of the different populations of neurons, measured not only over the duration of SSA as a whole but also over each of the active epochs of the oscillatory activity. This allowed us to extract the global silent epochs from the statistics, making the comparison between different cases more accurate. In fact, measurements of individual frequencies of the neurons confirmed that the active individual neurons shared the leading frequency with the whole module they belonged to, and only the weakly active neurons (with a firing rate of a few Hz) fired independently (not shown).

Similarly to the firing rate of excitatory RS neurons, when 20% of all excitatory neurons were of the CH type the firing rate of the inhibitory neurons (both of the LTS or FS types) doubled, and when the proportion of CH neurons reached 40% the firing rate of these inhibitory neurons tripled. This can be seen directly from the columns in Table 3 representing the corresponding firing rates. The presence (both of 20% or 40%) of CH neurons in the network did not alter the tendency described above of greater uniformity in the distribution of firing rates of the two types of inhibitory neurons: the mean firing rate and the corresponding maximal firing rate of the FS neurons was higher than for the LTS neurons; however, the median of the firing rate distribution was still lower for FS neurons than for LTS neurons (see Table 3). This again meant presence of a few very active FS inhibitory neurons on one side of the distribution and of many weakly active FS neurons on its other side. In comparison, most of the LTS neurons were active with moderate firing rates.

The effect of introducing excitatory neurons of the IB type in the network was not as notable on the firing rates of inhibitory neurons (both of LTS or FS types) as the effect of CH excitatory neurons but nevertheless networks with IB excitatory neurons displayed small increments in the firing rates of their inhibitory neurons, which were stronger for 40% than for 20% of IB neurons. The same ocurred with the total excitation and inhibition produced by the network, as can be seen from Table 3.

Finally, and also akin to the firing rate of RS excitatory neurons, the effect of modularity on the activity measures shown in Table 3 was not so strong. For non-zero hierarchical levels, the total inhibition and excitation produced by a network and the firing rate of its inhibitory neurons with otherwise fixed neuron types remained in the same range as for a network with *H* = 0. The same was accordingly true for the distributions of the firing rates of the different types of inhibitory neurons (not shown). Difference in total excitation and inhibition was also not strongly influenced by merely exchanging the type of inhibitory neurons and keeping all other network parameters fixed (see Table 3).

## 4. Discussion

We have constructed a spiking network model that captures elements of the architectonic organization of the cortex and of its composition in terms of cells of different electrophysiological classes. The architecture of the network is hierarchical and modular, which arguably (Wang et al., 2011; Samu et al., 2014) represents the generic topological organization of the cortex across many spatial scales, and the excitatory and inhibitory cells of our model belong to five distinct electrophysiological classes that can coexist in the same network (Nowak et al., 2003; Contreras, 2004). Our goal was to study the combined effect of these architectonic and physiological elements on the SSA of the network. To do so we performed an extensive computational study of our model by considering network architectures characterized by different combinations of hierarchical and modularity levels, mixture of excitatory-inhibitory neurons, strength of excitatory-inhibitory synapses and network size submitted to distinct initial conditions.

Our main finding is that the neuronal composition of the network, i.e., the types and combinations of excitatory and inhibitory cells that comprise the network, has an effect on the properties of SSA in the network, which acts in conjunction with the effect of network topology. Previous theoretical studies have emphasized the role of the structural organization (topology) of the cortical network on its sustained activity (Kaiser and Hilgetag, 2010; Wang et al., 2011; Garcia et al., 2012; Litwin-Kumar and Doiron, 2012; Potjans and Diesmann, 2014). Here we have shown that the electrophysiological classes of the cortical neurons and the percentages of these neurons in the network composition also affect the dynamics of the sustained network activity. Specifically, we found that networks comprising excitatory neurons of the RS and CH types have higher probability of supporting long-lived SSA than networks with excitatory neurons only of the RS type. In addition, the type of the inhibitory neurons in the network also has a significant effect. In particular, LTS inhibitory neurons stronger favor long-lived SSA states than FS inhibitory neurons.

A possible mechanism that would render networks made of RS and CH excitatory cells more prone to long-lived SSA is due to the pattern of spikes exhibited by the CH cells, which consists of spike bursts followed by strong afterhyperpolarizations. The presence of CH neurons in the network would then enhance and coordinate the postsynaptic responses of other network cells, which would contribute to prolongation of network actredivity. As a consequence, the global network activity would become more oscillatory and better synchronized with corresponding increases in the global network frequency and the mean firing frequency of the individual neurons, effects reported in Section 3. This mechanism is more effective in networks with inhibitory neurons of the LTS class rather than of the FS class because of the higher temporal and spatial uniformity of the inhibition provided by LTS neurons, as discussed in Section 3.4.

We are aware of just one theoretical study in the literature which has addressed the impact of the specific neuronal composition of the network on its SSA regimes (Destexhe, 2009). There, it was shown that a two-layered cortical network in which the layers were composed of excitatory RS and inhibitory FS cells with a small proportion of excitatory LTS cells in the second layer, could produce SSA. Here we have extended the analysis by including neurons of five electrophysiological classes and, in particular, by considering LTS cells that are exclusively inhibitory.

Our study also has shown that modularity favors SSA. In general, independently of neuronal composition, the increase in the hierarchical level of the network (and hence in the number of modules) increases the lifetime expectancy of SSA in the network. This effect can be understood if we imagine that distinct modules are activated intermittently and non-simultaneously. Each module is a random network which, depending on its specific neuronal composition, can generate SSA with a certain lifetime. Because of the sparse coupling among modules, they activate each other in an alternate way so that there is a probability of each one of them activating a neighbor before decaying to rest. And the larger the number of modules, the greater is this probability.

The region of the parameter space of excitatory and inhibitory synaptic strengths for which the network SSA states display properties similar to physiological measurements (Softky and Koch, 1993; Hromádka et al., 2008; Maimon and Assad, 2009; Haider et al., 2013) is the lower right corner of what we called the diagram of low synaptic strengths. The spiking properties of the SSA states in this region are remarkably independent of the network architecture and initial conditions. These properties are irregular neuronal firing and low frequency population oscillation with leading frequency often in the range of ~5 to ~8 Hz. In this particular region of the (*g*_ex_, *g*_in_) plane the ratio *g*_ex_/*g*_in_ has a value between about 4 and 12. This is consistent with the theoretical prediction that irregular activity in a spiking cortical network can be sustained in a balanced excitation-inhibition state whereby the strength of inhibitory synapses is higher than the strength of excitatory synapses to compensate for the smaller number of inhibitory neurons, and keep the average total synaptic input into a neuron near zero, so that the neuron spikes are caused by the fluctuations around this average (van Vreeswijk and Sompolinsky, 1996; Amit and Brunel, 1997; van Vreeswijk and Sompolinsky, 1998; Brunel, 2000). These theoretical studies relied on random networks of sparsely-connected leaky integrate-and-fire neurons. Our study, although more focused on hierarchical and modular networks, also has shown that irregular SSA can occur in random networks (*H* = 0). Since our networks are based on neuron models with richer properties than the leaky integrate-and-fire model, our finding points to a complementary, though secondary in comparison with the excitation-inhibition balance, mechanism for irregular SSA in a random network of spiking neurons, which depends on the mixture and proportions of the different types of excitatory and inhibitory neurons in the network.

Our results strongly suggest that the sustained and irregular firing regimes in our simulations are chaotic. This is consistent with conjectures that the default state of the brain is chaotic (Skarda and Freeman, 1987; van Vreeswijk and Sompolinsky, 1996, 1998; Banerjee et al., 2008; Izhikevich and Edelman, 2008; London et al., 2010). It is important to note that in the biologically relevant range of low synaptic strengths the SSA does not last indefinitely: its lifetime remains finite and abruptly ends with relaxation toward the state of rest. The probability to observe a SSA of a given duration is an exponential function of duration. From this point of view, SSA is a transient phenomenon. In a way, this was already expected because every brain dynamical regime is transient (Rabinovich and Varona, 2012). Duration of the transient depends on the network architecture (hierarchical level, mixture of excitatory-inhibitory neurons) and the synaptic parameters. A direct possibility to prolong the lifetime of the SSA without increasing the synaptic strengths is to increase the number of neurons, since the escape time of transient chaotic trajectories grows exponentially with the dimension of the system (Crutchfield and Kaneko, 1988; Kumar et al., 2008; El Boustani and Destexhe, 2009; Lai and Tél, 2011). We observed this effect when proceeding from 2^9^ to 2^10^ neurons; our preliminary results with larger networks confirm this conjecture.

### Conflict of interest statement

The authors declare that the research was conducted in the absence of any commercial or financial relationships that could be construed as a potential conflict of interest.
